# 

**Published:** 1901-01

**Authors:** 


### The Medical Societies.

THE CENTRAL TEXAS MEDICAL ASSOCIATION held its semi-annual session at Fort Worth January 8 and 9 inst. There was a very good attendance and some valuable papers were read. Dr. Jelks, of Hot Springs, Arkansas, attended the meeting and contributed a paper. The *Journal* has not, as yet, been favored with a report of the proceedings—nor any of the papers. We are gratified to learn that Dr. N. A. Olive, the long time courteous, popular and efficient Secretary was elected President.

### Medical Association of Texas, New Mexico, Arizona and Mexico.

First meeting will be held Thursday, Friday, Saturday, January 17, 18, 19, 1901, K. of P. Hall, Turner Building, El Paso, Texas.

Special rates on railroads and at hotels.

Program received too late for publication.

*Committee of Arrangements*—Drs. W. N. Vilas, S. T. Turner and F. W. Gallagher.

